# Maternal age at childbirth and the risk of attention-deficit/hyperactivity disorder and learning disability in offspring

**DOI:** 10.3389/fpubh.2023.923133

**Published:** 2023-02-02

**Authors:** Ling Gao, Songtao Li, Yulin Yue, Guangfeng Long

**Affiliations:** Department of Clinical Laboratory, Children's Hospital of Nanjing Medical University, Nanjing, China

**Keywords:** maternal age, attention-deficit/hyperactivity disorder, learning disability, NHANES, epidemiology

## Abstract

**Background:**

Studies have shown that young maternal age at childbirth can increase the risk of attention-deficit/hyperactivity disorder (ADHD) in offspring, but a study of the U.S. population has not been reported. Moreover, there is no reported research on young and advanced maternal age at childbirth and whether it can contribute to the risk of learning disability (LD) in offspring.

**Methods:**

This study evaluated the association between young and advanced maternal age at childbirth and offspring risk of ADHD and LD in the U.S. population. Using data from 8,098 participants included in the National Health and Nutrition Examination Survey (NHANES) conducted in 1999–2004, we analyzed the association between maternal age at childbirth and ADHD and LD risk in offspring. Odds ratios (ORs) and 95% confidence intervals (CIs) for maternal age at childbirth in association with ADHD and LD risk in offspring were estimated using multivariate logistic regression models after adjustment for age, sex, race, body mass index (BMI), poverty income ratio, smoking status during pregnancy, and NHANES cycle. Restricted cubic spline (RCS) models were used to evaluate potential non-linear relationships. Sensitivity analyses were performed to ensure the reliability of the results.

**Results:**

Among all participants, the offspring of subjects with a maternal age at childbirth of 18–24 years had an increased risk of ADHD (OR = 1.34, 95% CI: 1.01, 1.79) and LD (OR = 1.36, 95% CI: 1.06, 1.79) or either ADHD or LD (OR = 1.48, 95% CI: 1.20, 1.81). Additionally, compared with subjects with a maternal age at childbirth of 25–29 years, subjects with a maternal age at childbirth of 35–39 years had lower odds of having offspring with ADHD (OR = 0.60, 95% CI: 0.36, 1.00) and higher odds of having offspring with LD (OR = 1.34, 95% CI: 1.01, 1.78). The relationship between maternal age at childbirth and LD risk presented a *U*-shaped curve.

**Conclusions:**

These results provide epidemiological evidence showing that young and advanced maternal age at childbirth are associated with ADHD and LD risk.

## Introduction

Maternal age at childbirth is defined as the age of the mother at the time of delivery. Generally, a young maternal age at childbirth is defined as an age of 19 years old or younger, and an advanced maternal age is defined as an age of at least 35 years old (1). The effects of young and advanced maternal age at childbirth may affect the health outcomes of mothers and their offspring. Previous studies have suggested that a young maternal age at childbirth is associated with maternal cardiovascular disease risk (2) and low birth weight, preterm birth, and neonatal mortality (3). Several adverse health effects are associated with advanced maternal age at childbirth, including cerebral palsy, neurocognitive disorders, psychiatric disorders (4), stillbirth (5–7) and maternal cancer risk (8, 9); however, some studies have reported that advanced maternal age does not seem to be associated with the long-term morbidity of offspring (10).

Neurological disorders in children manifest as abnormalities in neurocognition, self-regulation, and adaptive functioning (11). Abnormal neurobehavioral function in children can cause health problems and other issues, such as emotional disorders (12), lower education levels (13), antisocial behavior (14), and premature mortality (15). Among neurological disorders, attention-deficit/hyperactivity disorder (ADHD) and learning disability (LD) are common in children. ADHD and LD are defined as persistent inattention and hyperactivity and a reduced ability to learn, respectively. The global prevalence of ADHD in children is ~5% (16), and the prevalence is reported to be 18.1% in Tunisian adolescents (17), 8.8% in Nigerian children (18), and 6.26% in Chinese children and teenagers (19). The prevalence rates of LD in the U.S. and in a city in India were shown to be 9.7 (20) and 3.08% (21), respectively. Previous studies on the risk factors for ADHD and LD have mainly focused on genetic factors (15, 22–24), while ~10–40% of the risk may be related to factors such as premature birth and smoking during pregnancy for ADHD (25, 26) and low birth weight (27), maternal BMI before pregnancy (28), and maternal anemia (29) for LD. Although many previous studies have suggested that ADHD is highly related to hereditary factors, no gene with a definite pathogenic effect has been identified (30). Although Thapar et al. (15) found that both prenatal and perinatal factors may be risk factors for ADHD and LD, these risk factors are not fully understood. Therefore, fully understanding these risk factors may effectively prevent the occurrence of ADHD and LD.

A recent study found that a young maternal age at childbirth was positively correlated with the risk of ADHD in offspring in Denmark (31). However, there is a lack of relevant research in the U.S. Because immigrants from multiple countries live in the U.S., the genetic background and cultural differences of the population are different from those of Denmark. Additionally, the prevalence of ADHD in the U.S. is much higher than the average level globally (32). The average maternal age at first childbirth increased from 1970 to 2006 and from 2011 to 2012 in the U.S. (33). This study was performed due to the lack of U.S. research on the association between maternal age at childbirth and ADHD and LD risk in offspring. This study is the first to explore the association between maternal age at childbirth and ADHD and LD in offspring and to evaluate the non-linear relationship in the U.S.

## Methods

### Study population

**Figure 1:**
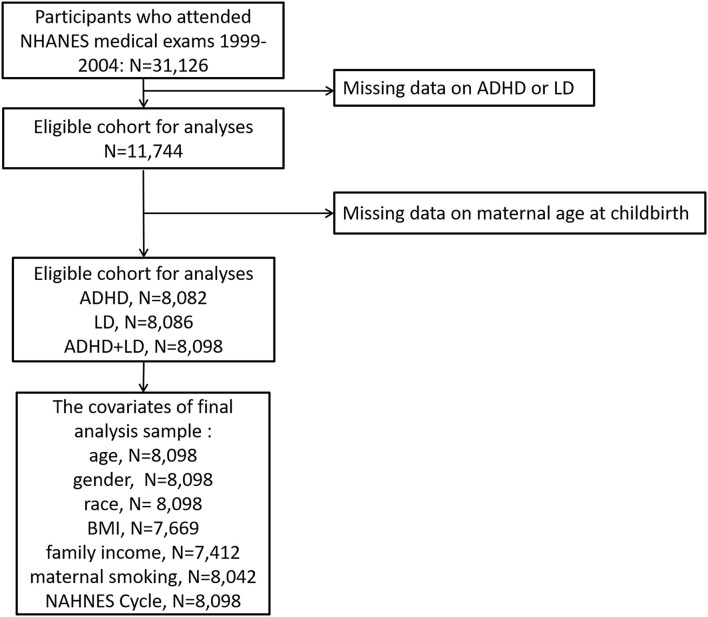
Eligible participants and those included in the analyses of the associations between maternal age at childbirth and the risk of ADHD and LD in offspring in the U.S. population.

The National Health and Nutrition Examination Survey (NHANES) is conducted by the National Center for Health Statistics (NCHS) at the Centers for Disease Control and Prevention every 2 years, and the main purpose is to assess the health and nutritional status of children and adults in the U.S. The user data agreement is available online (https://www.cdc.gov/nchs/data\_access/restrictions.htm). The NCHS Research Ethics Review Board approved these NHANES cycles. All subjects provided written informed consent. Data on ADHD and LD outcomes are available only for the 1999–2004 cycle; thus, we obtained publicly available NHANES data generated through surveys conducted in 1999–2004, including data from a total of 31,126 participants. By using unique survey participant identifiers, we combined information on the participants' characteristics with their questionnaire information. For maternal age at childbirth, data are available only for offspring aged 0–15 years, and the age ranges for ADHD and LD evaluation were 4–19 and 4–15 years, respectively. We therefore excluded participants under 4 and over 15 years of age. Although pregnancy may affect the outcomes of analysis, we did not exclude pregnant subjects (*n* = 7) in our analysis. Ultimately, 8,098 subjects were included in our study: 3,977 boys and 4,121 girls. The flow chart for inclusion and exclusion is shown in Figure 1.

### Assessment of maternal age at childbirth and ADHD and LD risk

This information was based on parental/guardian responses to the following NHANES interview question, asked twice regarding both LD and ADHD (for children ≤ 15 years of age): “How old was your biological mother when you were born?” Age was recorded as a continuous variable. The outcomes of ADHD and LD were obtained from parental reports of LD and/or ADHD. Specifically, the data were based on parental/guardian responses to two NHANES interview questions: “Has a doctor or health professional ever told you that you have attention deficit disorder?” and “Has a representative from a school or a health professional ever told you that you have a learning disability?” For children ≥4 years old, the same questions were asked regarding both LD and ADHD. The answer to both of these questions was “yes” or “no.” The answer was included as a binary variable for subsequent analysis. We divided children into those with parental/guardian reports of ADHD, those with parental/guardian reports of LD, and those with parental/guardian reports of either LD or ADHD.

### Covariates

Several factors related to outcomes were adjusted in our logistic regression analysis, including age (continuous variable), sex (categorical variable), race (categorical variable), body mass index (BMI, continuous variable), poverty income ratio (PIR, categorical variable), and NHANES cycle (categorical variable). Information regarding age, sex, race, and the PIR was obtained by questionnaires conducted during face-to-face interviews. Height and body weight were recorded in a physical examination and were used to calculate BMI. Information related to smoking during pregnancy was based on parental/guardian responses to the following question: “Did your biological mother smoke at any time while she was pregnant?”

### Statistical methods

Continuous variables are presented as the mean and standard deviation, and categorical variables are presented as the frequency and percentage. Because BMI and PIR values were missing for 429 and 686 participants, respectively, to ensure that the sample size was not reduced, we used the mean value imputation method. We divided participants into the following groups according to maternal age at childbirth: ≤ 17, 18–24, 25–29, 30–34, 35–39, and ≥40 years age groups. Considering that both young and advanced maternal age at childbirth may be risk factors for ADHD or LD, we regarded 25–29 years as the reference age (34). Logistic regression was used to assess the association between maternal age at childbirth and ADHD, LD and either ADHD or LD risk after adjusting for age, sex, race, BMI, PIR, smoking during pregnancy, and NHANES cycle. In addition, we used restricted cubic spline (RCS) regression to investigate the non-linear trend between maternal age at childbirth and the risk of ADHD, LD and either ADHD or LD. For the sensitivity analysis, we excluded subjects without BMI or PIR data in logistic regression analysis. *P* < 0.05 was considered a statistically significant difference. In multiple comparisons, to avoid false positives, the Bonferroni correction was used in our analysis, with *P*-values < (0.05/5 [group] = 0.01) considered statistically significant. Stata version 14.0 (StataCorp) was used for the statistical analyses in the present study.

## Results

The average maternal age at childbirth in our study was 25.6 ± 6.1 years old (Table 1). Maternal age at childbirth differs among races; this age was the lowest in the non-Hispanic Black population (24.5 ± 6.1 years old) and the highest in the non-Hispanic White population (27.3 ± 5.8 years old). On average, subjects with high family income had a higher maternal age at childbirth than those with low family income (27.4 ± 5.8 vs. 24.0 ± 6.0 years old, *P* < 0.001). In addition, maternal age at childbirth did not differ significantly on the basis of smoking during pregnancy (25.6 ± 6.0 vs. 25.6 ± 6.1 years old, *P* = 0.961).

**Table 1:** Characteristics of maternal age of children included in the study (NHANES 1999–2004; *n* = 8,098).

**Characteristic**	***N* (%)**	**Mean ±SD**	***P*-value**
Overall	8,098 (100.0%)	25.6 ± 6.1	
**Sex**			0.044
Boy	3,977 (49.1%)	25.4 ± 6.1	
Girl	4,121 (50.9%)	25.7 ± 6.1	
**Race/ethnicity**			<0.001
Mexican American	2,700 (33.3%)	25.1 ± 5.8	
Other hispanic	347 (4.3%)	25.9 ± 6.5	
Non-hispanic white	2,168 (26.8%)	27.3 ± 5.8	
Non-hispanic black	2,541 (31.4%)	24.5 ± 6.1	
Other race—including multi-racial	342 (4.2%)	26.5 ± 6.2	
**Smoking during pregnancy**			0.961
Yes	1,162 (14.3%)	25.6 ± 6.0	
No	6,880 (85.0%)	25.6 ± 6.1	
**Age (years)**			0.002
4–7	2,303 (28.4%)	26.0 ± 6.3	
8–11	2,217 (27.4%)	25.5 ± 6.0	
12–15	3,578 (44.2%)	25.4 ± 6.0	
**NHANES cycle**			0.028
1999–2000	2,691 (33.2%)	25.3 ± 6.0	
2001–2002	2,874 (35.5%)	25.8 ± 6.1	
2003–2004	2,533 (31.3%)	25.6 ± 6.2	
**PIR**			<0.001
<1.08	2,711 (33.5%)	24.0 ± 6.0	
1.08–2.17	2,700 (33.3%)	25.3 ± 6.0	
>2.17	2,687 (33.2%)	27.4 ± 5.8	

Table 2 shows that the percentages of offspring with ADHD, LD and either ADHD or LD in our study were 6.4, 10.4, and 13.5%, respectively. The maternal age at childbirth for the group with ADHD, LD, or either ADHD or LD was younger than that for the group without ADHD, LD, or either ADHD or LD (25.2 vs. 26.7 years old, *P* = 0.001 for ADHD; 25.7 vs. 26.6 years old, *P* < 0.001 for LD; 25.5 vs. 26.7 years old, *P* < 0.001 for either ADHD or LD).

**Table 2:** Mean maternal age at childbirth based on outcomes for study participants (*n* = 8,098).

**Neurodevelopmental outcome**	***N* (%)**	**Mean (95% CI)**	***P*-value**
**ADHD**			0.001
Yes	521 (6.4%)	25.2 (24.3, 26.0)	
No	7,561 (93.6%)	26.7 (26.3, 27.0)	
**LD**			<0.001
Yes	841 (10.4%)	25.7 (25.2, 26.3)	
No	7,245 (89.6%)	26.6 (26.3, 27.0)	
**Either ADHD or LD**			<0.001
Yes	1,090 (13.5%)	25.5 (24.9, 26.2)	
No	7,008 (86.5%)	26.7 (26.4, 27.0)	

Weighted mean.

ADHD, attention-deficit/hyperactivity disorder; LD, learning disability.

The logistic regression results showed that compared to subjects aged 25–29 years, in subjects younger than 25 years old and older than 39 years old at childbirth, the risk of ADHD, LD, and either ADHD or LD in offspring was increased (Table 3) after adjusting for age, sex, race, BMI, PIR, smoking during pregnancy, and NHANES cycle. Among these groups, the adjusted OR for subjects aged 18–24 years was 1.48 (95% CI: 1.20, 1.81) for the risk of either ADHD or LD in offspring, with a *P*-value < 0.01 (Bonferroni adjustment of the *P*-value). In addition, we also observed that compared to subjects aged 25–29 years at childbirth, in subjects aged 30–39 years, the risk of ADHD was reduced, while the risk of LD and the risk of either ADHD or LD in offspring was increased. However, these results were not statistically significant, which may be related to our small sample size. The sensitivity analysis showed that the results were robust (Supplementary Table S1).

**Table 3:** Association between maternal age at childbirth and parent-reported LD, ADHD, and either LD or ADHD.

		** ≤ 17**	**18–24**	**25–29**	**30–34**	**35–39**	**≥40**
ADHD	Model 1	1.08 (0.72, 1.62)	1.38 (1.04, 1.84)	1	0.93 (0.61, 1.43)	0.56 (0.34, 0.93)	1.30 (0.49, 3.48)
	Model 2	1.15 (0.75, 1.78)	1.39 (1.04, 1.84)	1	0.95 (0.62, 1.46)	0.58 (0.35, 0.97)	1.34 (0.50, 3.55)
	Model 3	1.04 (0.68, 1.60)	1.34 (1.01, 1.79)	1	0.99 (0.63, 1.53)	0.60 (0.36, 1.00)	1.48 (0.56, 3.93)
LD	Model 1	2.05 (1.30, 3.25)	1.58 (1.20, 2.08)	1	1.23 (0.85, 1.78)	1.18 (0.90, 1.56)	1.98 (0.80, 4.92)
	Model 2	1.69 (1.07, 2.67)	1.39 (1.06, 1.83)	1	1.35 (0.94, 1.95)	1.31 (0.99, 1.74)	2.34 (0.97, 5.70)
	Model 3	1.61 (0.99, 2.62)	1.36 (1.02, 1.79)	1	1.41 (0.96, 2.05)	1.34 (1.01, 1.78)	2.59 (1.05, 6.35)
Either ADHD or LD	Model 1	1.88 (1.35, 2.61)	1.63 (1.34, 1.98)	1	1.12 (0.81, 1.55)	1.05 (0.80, 1.39)	1.91 (0.86, 4.24)
	Model 2	1.72 (1.23, 2.39)	1.52 (1.25, 1.85)	1	1.18 (0.86, 1.63)	1.12 (0.84, 1.50)	2.13 (0.98, 4.63)
	Model 3	1.59 (1.12, 2.28)	1.48 (1.20, 1.81)^*^	1	1.23 (0.89, 1.70)	1.15 (0.85, 1.54)	2.35 (1.09, 5.09)

Model 1: adjusted for age and sex.

Model 2: adjusted for age, sex, race, BMI, and PIR.

Model 3: adjusted for age, sex, race, BMI, PIR, smoking during pregnancy, and NHANES cycle.

^*^The P-value was less than the Bonferroni adjustment (0.010).

**Figure 2:**
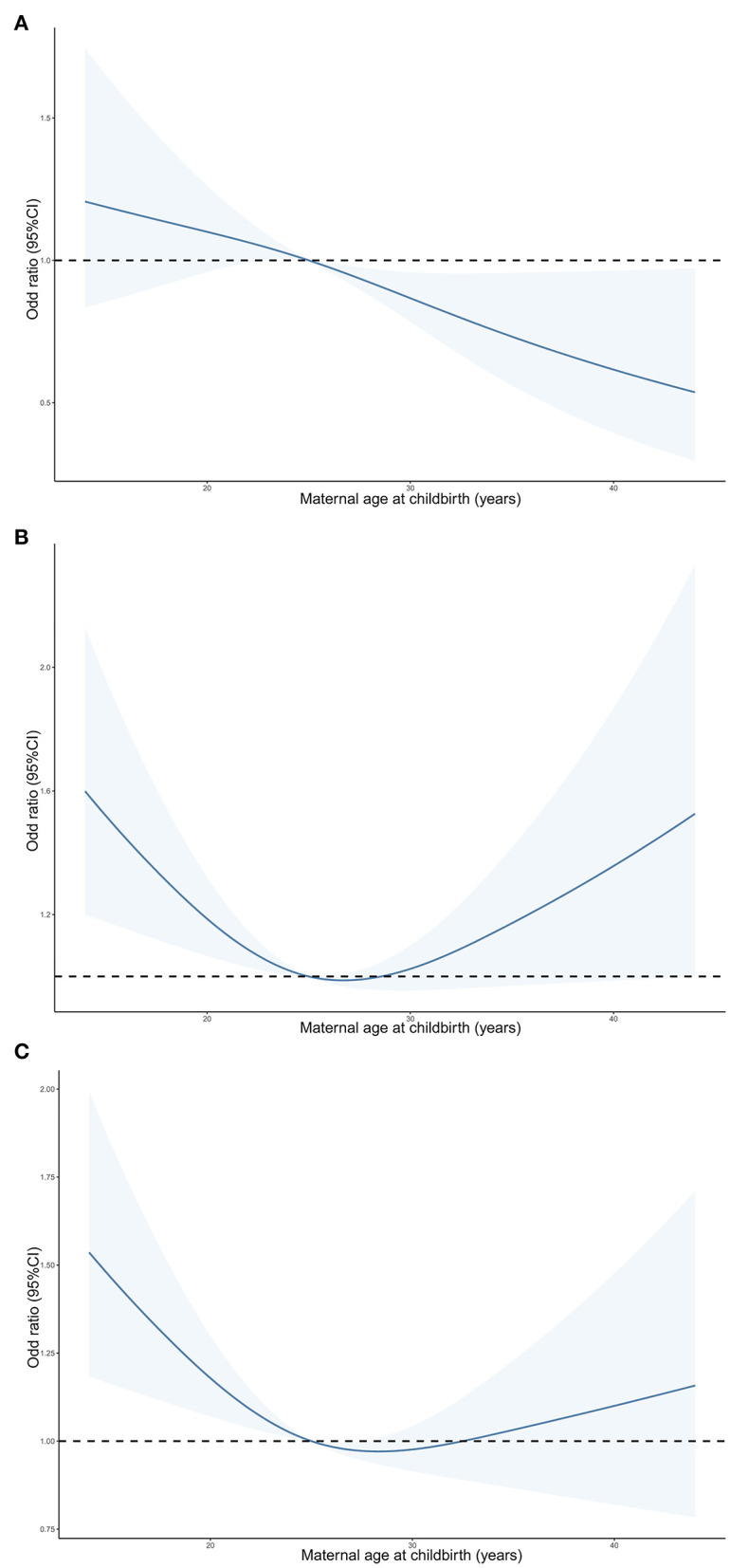
Predicted spline curves for the associations of maternal age at childbirth with the risk of ADHD **(A)**, LD **(B)** and either ADHD or LD **(C)** in offspring according to RCS regression models. ADHD, attention-deficit/hyperactivity disorder; LD, learning disability.

When maternal age at childbirth was considered as a continuous variable using RCS regression models (Figure 2), a *U*-shaped association of maternal age at childbirth with the risk of either ADHD or LD in offspring was observed (*P*-value = 0.007). The risk of either ADHD or LD in offspring was lowest among subjects aged 25–29 years at childbirth. When performing RCS regression models of the LD risk outcome, we observed a similar but more pronounced *U*-shaped curve with a greater slope (*P*-value = 0.005). However, the non-linear association between maternal age at childbirth and ADHD risk in offspring was not significant (*P*-value = 0.842).

## Discussion

This is one of the first studies to report that young maternal age at childbirth may increase the risk of ADHD and LD in offspring in the U.S. population and that advanced maternal age at childbirth seems to likely increase the risk of LD in offspring. In addition, we also found that these associations differ by sex. Furthermore, the relationship between maternal age at childbirth and the risk of LD in offspring presents a *U*-shaped curve.

Previous epidemiological studies have shown an increased risk of ADHD in the offspring of mothers with young maternal age at childbirth in the Swedish population (35); similarly, the risk of ADHD in offspring is also increased for mothers with young maternal age at childbirth in the Danish population (31). The results of the above two studies are similar to ours and may be explained because the development of ADHD is related to neurotransmission pathways. Therefore, we speculated that the education levels of the subjects with advanced maternal age at childbirth were higher, and these individuals may have greater health knowledge during pregnancy. Therefore, compared to participants in the 25–29-year-old group, participants in advanced maternal age groups may pay more attention to nutritional supplements and additives during pregnancy, such as folic acid, which are beneficial for neural development, thus reducing the risk of ADHD in their offspring. Other potential reasons are that individuals in the U.S. population have complex ethnic backgrounds. The genetic backgrounds of different races are quite different, and genetic factors are considered to be related to the development of ADHD (36, 37). In contrast to the results of the present study, a study of the Danish population revealed that advanced maternal age at childbirth (over 35 years old) is a protective factor against ADHD in offspring. While our results showed that the risk of ADHD in offspring of individuals older than 39 years is likely to be increased, the difference was not significant, which may be related to the small sample size of our study. Another reason for the difference in study results is that the Danish study did not group and analyze the data of people over 40 years old.

At present, few studies have been conducted on maternal age at childbirth and LD risk in offspring, which may be related to the diagnosis of LD without International Classification of Disease (ICD) standards. Although LD evaluation is subjective, it is easy to perform and can reflect certain neural functions (38, 39). At present, the etiology of LD is still unclear. Some scholars have suggested that genetic factors may play a role, and the symptoms of LD are also broader. Adolescents who exhibit abnormalities in reading, writing, listening, speaking, and performing math are suggested to have LD. However, the cause of LD in offspring is complex and may not be related to a neural pathway. In this study, we observed a *U*-shaped curve of maternal age at childbirth and offspring risk of LD. It is suggested that the offspring of individuals with younger and more advanced maternal ages at childbirth have a greater risk of LD. At present, most of the existing studies suggest that young maternal age at childbirth increases the risk of health effects on offspring. However, controversy exists for subjects with advanced maternal age at childbirth. An appropriate older maternal age is beneficial for offspring with regard to financial status and education level (1). The social economic position and resources of older mothers are greater than those of younger mothers, and over time, the offspring of older mothers experience the effects of improvements in public health conditions and educational expansion. Although negative effects occur during the perinatal period, there is no difference between the offspring of older mothers and those of younger mothers during adulthood (40). However, it should be noted that the oocytes of older mothers are aged, and accumulated DNA damage (41) and the imbalance in mitochondrial homeostasis (42) in aged oocytes have a negative impact on the development of fertilized eggs (43). DNA damage and mitochondrial function are closely related to nerve development (44, 45). These characteristics of aged oocytes may explain the decreased learning ability of offspring. However, some researchers suggest that some patients are classified as having an LD because of their parents' misunderstanding of the education process and higher parental expectations for offspring (46). The intelligence or neurodevelopment of offspring may not be abnormal, but the specific molecular mechanism of the relationship between age and LD risk still requires further research.

Our results indicate that maternal age at childbirth is related to the risk of LD in offspring. Due to the recall bias present in retrospective cohorts, animal studies may clarify the cause and effect relationship. Some existing mechanistic studies have confirmed that the spatial learning capacity of mice at 32–35 weeks is lower than that of mice at 9–12 weeks, which may be related to the decrease in the expression of vitamin D receptor (VDR) in the early embryogenesis process (47). In addition, compared with 3-month-old mice, 15–18-month-old mice showed anxiety-like behavior, and the gene expression pattern of the hippocampus was also changed (48). We also speculated that late production age may be related to egg quality (49). These possible mechanisms may explain the link between maternal age at childbirth and ADHD and LD risk in offspring. However, there are few studies on the mechanism of the relationship between young maternal age at childbirth and ADHD and LD risk in offspring, possibly because few animal models exist. We speculated that the link between young maternal age at childbirth and ADHD and LD risk in offspring may arise because the family income and education levels of young mothers are lower than those of older mothers (50). Additionally, Fall et al. found that young maternal age at childbirth may be associated with poor birth outcomes and nutrition for offspring in low- and middle-income countries (1).

As an advantage, this study is the first to report the association between maternal age at childbirth and offspring ADHD and LD risk in the U.S. population. We found that young or advanced maternal age at childbirth may lead to increased ADHD and LD risk in offspring. Through the results of subgroup analysis, we identified the susceptible population, which may provide a basis for research on the underlying mechanism or prevention. Applying the RCS model is helpful to determine the association between maternal age at childbirth and the risk of ADHD and LD in offspring.

Although the present study results have some scientific significance, there are still several limitations of our study as a whole. First, genetic factors have a great influence on ADHD and LD risk. Even if we consider race as a covariate for adjustment and further analysis, we still cannot exclude the bias of genetic factors. Second, some maternal lifestyles during pregnancy also affect offspring ADHD and LD risk, although we adjusted for the variable of smoking during pregnancy. In fact, there are additional factors that may affect ADHD and LD risk in offspring, such as gestational hypertension and gestational diabetes, but we could not adjust for them because no such data are contained in the NHANES database. Third, in this study, some covariates were missing. To avoid reducing the sample size, we imputed data for the missing variables, but strictly speaking, this approach may have biased the actual results. Fourth, the outcome variables in this study were based on self-reported data, leading to potential subjective bias. It is thus necessary for future research to use objective indicators.

In conclusion, the present study results indicated a *U*-shaped association between maternal age at childbirth and the risk of ADHD and LD in offspring in a nationally representative U.S. survey. Future studies with large sample sizes and mechanistic studies are needed to confirm the risk of ADHD and LD in offspring.

## Data availability statement

The datasets presented in this study can be found in online repositories. The names of the repository/repositories and accession number(s) can be found below: NHANES.

## Ethics statement

The studies involving human participants were reviewed and approved by the NCHS Research Ethics Review Board approved these NHANES cycles. The patients/participants provided their written informed consent to participate in this study. Written informed consent was obtained from the individual(s) for the publication of any potentially identifiable images or data included in this article.

## Author contributions

Material preparation, data processing, analysis were carried out, and the first draft of the manuscript was written by GL. All authors commented on the previous versions of the manuscript, contributed to the conception and design of the study, read, and approved the final manuscript.

## References

[1] Fall CH Sachdev HS Osmond C Restrepo-Mendez MC Victora C Martorell R. Association between maternal age at childbirth and child and adult outcomes in the offspring: a prospective study in five low-income and middle-income countries (COHORTS collaboration). Lancet Global Health. (2015) 3:e366–77. 10.1016/S2214-109X(15)00038-8 25999096 PMC4547329

[2] Rosendaal NTA Pirkle CM. Age at first birth and risk of later-life cardiovascular disease: a systematic review of the literature, its limitation, and recommendations for future research. BMC Public Health. (2017) 17:627. 10.1186/s12889-017-4519-x 28679414 PMC5498883

[3] Gibbs CM Wendt A Peters S Hogue CJ. The impact of early age at first childbirth on maternal and infant health. Paediat Perinat Epidemiol. (2012) 26:259–84. 10.1111/j.1365-3016.2012.01290.x 22742615 PMC4562289

[4] Balasch J Gratacos E. Delayed childbearing: effects on fertility and the outcome of pregnancy. Curr Opin Obstet Gynecol. (2012) 24:187–93. 10.1097/GCO.0b013e3283517908 22450043

[5] Lean SC Derricott H Jones RL Heazell AEP. Advanced maternal age and adverse pregnancy outcomes: a systematic review and meta-analysis. PLoS ONE. (2017) 12:e0186287. 10.1371/journal.pone.0186287 29040334 PMC5645107

[6] Carolan M Frankowska D. Advanced maternal age and adverse perinatal outcome: a review of the evidence. Midwifery. (2011) 27:793–801. 10.1016/j.midw.2010.07.006 20888095

[7] Carolan M. Maternal age ≥45 years and maternal and perinatal outcomes: a review of the evidence. Midwifery. (2013) 29:479–89. 10.1016/j.midw.2012.04.001 23159159

[8] Merrill RM Fugal S Novilla LB Raphael MC. Cancer risk associated with early and late maternal age at first birth. Gynecol Oncol. (2005) 96:583–93. 10.1016/j.ygyno.2004.11.038 15721398

[9] Nassar AH Usta IM. Advanced maternal age. Part II: long-term consequences. Am J Perinatol. (2009) 26:107–12. 10.1055/s-0028-1090593 19021096

[10] Pariente G Wainstock T Walfisch A Sheiner E Harlev A. Advanced maternal age and the future health of the offspring. Fetal Diagn Therapy. (2019) 46:139–46. 10.1159/000493191 30466094

[11] Hagan JF Balachova T Bertrand J Chasnoff I Dang E Fernandez-Baca D. Neurobehavioral disorder associated with prenatal alcohol exposure. Pediatrics. (2016) 138:1553. 10.1542/peds.2015-1553 27677572 PMC5477054

[12] Lecendreux M Konofal E Faraone SV. Prevalence of attention deficit hyperactivity disorder and associated features among children in France. J Attent Disord. (2011) 15:516–24. 10.1177/1087054710372491 20679156

[13] Biederman J Faraone SV Spencer TJ Mick E Monuteaux MC Aleardi M. Functional impairments in adults with self-reports of diagnosed ADHD: a controlled study of 1001 adults in the community. J Clin Psychiatry. (2006) 67:524–40. 10.4088/JCP.v67n0403 16669717

[14] Marshal MP Molina BS. Antisocial behaviors moderate the deviant peer pathway to substance use in children with ADHD. J Clin Child Adolesc Psychol. (2006) 35:216–26. 10.1207/s15374424jccp3502_5 16597217 PMC2680090

[15] Thapar A Cooper M. Attention deficit hyperactivity disorder. Lancet. (2016) 387:1240–50. 10.1016/S0140-6736(15)00238-X 26386541

[16] Sayal K Prasad V Daley D Ford T Coghill D. ADHD in children and young people: prevalence, care pathways, and service provision. Lancet Psychiatry. (2018) 5:175–86. 10.1016/S2215-0366(17)30167-0 29033005

[17] Mhalla A Guedria A Brahem T Amamou B Sboui W Gaddour N. ADHD in tunisian adolescents: prevalence and associated factors. J Attent Disord. (2018) 22:154–62. 10.1177/1087054717702217 28381094

[18] Umar MU Obindo JT Omigbodun OO. Prevalence and correlates of ADHD among adolescent students in Nigeria. J Attent Disord. (2018) 22:116–26. 10.1177/1087054715594456 26220786

[19] Wang T Liu K Li Z Xu Y Liu Y Shi W. Prevalence of attention deficit/hyperactivity disorder among children and adolescents in China: a systematic review and meta-analysis. BMC Psychiatry. (2017) 17:32. 10.1186/s12888-016-1187-9 28103833 PMC5244567

[20] Altarac M Saroha E. Lifetime prevalence of learning disability among US children. Pediatrics. (2007) 119:S77–83. 10.1542/peds.2006-2089L 17272589

[21] Padhy SK Goel S Das SS Sarkar S Sharma V Panigrahi M. Prevalence and patterns of learning disabilities in school children. Indian J Pediat. (2016) 83:300–6. 10.1007/s12098-015-1862-8 26334861

[22] Banaschewski T Becker K Dopfner M Holtmann M Rosler M Romanos M. Attention-deficit/hyperactivity disorder. Deutsches Arzteblatt Int. (2017) 114:149–59. 10.3238/arztebl.2017.0149 28351467 PMC5378980

[23] Roberts JL Hovanes K Dasouki M Manzardo AM Butler MG. Chromosomal microarray analysis of consecutive individuals with autism spectrum disorders or learning disability presenting for genetic services. Gene. (2014) 535:70–8. 10.1016/j.gene.2013.10.020 24188901 PMC4423794

[24] Becker N Vasconcelos M Oliveira V Santos FCD Bizarro L Almeida RMM. Genetic and environmental risk factors for developmental dyslexia in children: systematic review of the last decade. Dev Neuropsychol. (2017) 42:423–45. 10.1080/87565641.2017.1374960 29068706

[25] Sciberras E Mulraney M Silva D Coghill D. Prenatal risk factors and the etiology of ADHD-review of existing evidence. Curr Psychiatry Rep. (2017) 19:1. 10.1007/s11920-017-0753-2 28091799

[26] Huang L Wang Y Zhang L Zheng Z Zhu T Qu Y. Maternal smoking and attention-deficit/hyperactivity disorder in offspring: a meta-analysis. Pediatrics. (2018) 141:2465. 10.1542/peds.2017-2465 29288161

[27] Johnson EO Breslau N. Increased risk of learning disabilities in low birth weight boys at age 11 years. Biol Psychiatry. (2000) 47:490–500. 10.1016/S0006-3223(99)00223-1 10715355

[28] Andersen CH Thomsen PH Nohr EA Lemcke S. Maternal body mass index before pregnancy as a risk factor for ADHD and autism in children. Eur Child Adolesc nt Psychiatry. (2018) 27:139–48. 10.1007/s00787-017-1027-6 28712019

[29] Wiegersma AM Dalman C Lee BK Karlsson H Gardner RM. Association of prenatal maternal anemia with neurodevelopmental disorders. JAMA Psychiatry. (2019) 18:1–12. 10.1001/jamapsychiatry.2019.2309 31532497 PMC6751782

[30] Gallo EF Posner J. Moving towards causality in attention-deficit hyperactivity disorder: overview of neural and genetic mechanisms. Lancet Psychiatry. (2016) 3:555–67. 10.1016/S2215-0366(16)00096-1 27183902 PMC4893880

[31] Hvolgaard Mikkelsen S Olsen J Bech BH Obel C. Parental age and attention-deficit/hyperactivity disorder (ADHD). Int J Epidemiol. (2017) 46:409–20. 10.1093/ije/dyw073 27170763

[32] Mahone EM Denckla MB. Attention-deficit/hyperactivity disorder: a historical neuropsychological perspective. J Int Neuropsychol Soc JINS. (2017) 23:916–29. 10.1017/S1355617717000807 29198277 PMC5724393

[33] Matthews TJ Hamilton BE. First births to older women continue to rise. NCHS Data Brief. (2014) 152:1–8. 24813228

[34] Sheen JJ Wright JD Goffman D Kern-Goldberger AR Booker W Siddiq Z. Maternal age and risk for adverse outcomes. Am J Obstet Gynecol. (2018) 219:390.e1–e15. 10.1016/j.ajog.2018.08.034 30153431

[35] Chang Z Lichtenstein P D'Onofrio BM Almqvist C Kuja-Halkola R Sjolander A. Maternal age at childbirth and risk for ADHD in offspring: a population-based cohort study. Int J Epidemiol. (2014) 43:1815–24. 10.1093/ije/dyu204 25355726 PMC4276066

[36] Faraone SV Asherson P Banaschewski T Biederman J Buitelaar JK Ramos-Quiroga JA. Attention-deficit/hyperactivity disorder. Nat Rev Disease Prim. (2015) 1:15020. 10.1038/nrdp.2015.20 27189265

[37] Tarver J Daley D Sayal K. Attention-deficit hyperactivity disorder (ADHD): an updated review of the essential facts. Child Care Health Dev. (2014) 40:762–74. 10.1111/cch.12139 24725022

[38] Whitsell LJ. Learning disorders as a school health problem. Neurological and psychiatric aspects. Calif Med. (1969) 111:433–45. 4902291 PMC1503720

[39] Erenberg G. Learning disabilities: an overview. Semin Neurol. (1991) 11:1–6. 10.1055/s-2008-1041198 2034911

[40] Barclay KaM M. Advanced maternal age and offspring outcomes: reproductive aging and counterbalancing period trends. Popul Dev Rev. (2016) 42:69–94. 10.1111/j.1728-4457.2016.00105.x

[41] Goldmann JM Seplyarskiy VB Wong WSW Vilboux T Neerincx PB Bodian DL. Germline de novo mutation clusters arise during oocyte aging in genomic regions with high double-strand-break incidence. Nat Genet. (2018) 50:487–92. 10.1038/s41588-018-0071-6 29507425

[42] May-Panloup P Boucret L Chao de la Barca JM Desquiret-Dumas V Ferre-L'Hotellier V Moriniere C. Ovarian ageing: the role of mitochondria in oocytes and follicles. Hum Reprod Update. (2016) 22:725–43. 10.1093/humupd/dmw028 27562289

[43] Miao YL Kikuchi K Sun QY Schatten H. Oocyte aging: cellular and molecular changes, developmental potential and reversal possibility. Hum Reprod Update. (2009) 15:573–85. 10.1093/humupd/dmp014 19429634

[44] McKinnon PJ. Genome integrity and disease prevention in the nervous system. Genes Dev. (2017) 31:1180–94. 10.1101/gad.301325.117 28765160 PMC5558921

[45] Khacho M Harris R Slack RS. Mitochondria as central regulators of neural stem cell fate and cognitive function. Nat Rev Neurosci. (2019) 20:34–48. 10.1038/s41583-018-0091-3 30464208

[46] Graham HR Minhas RS Paxton G. Learning problems in children of refugee background: a systematic review. Pediatrics. (2016) 137:3994. 10.1542/peds.2015-3994 27194628

[47] Mao WJ Wu ZY Yang ZH Xu YW Wang SQ. Advanced maternal age impairs spatial learning capacity in young adult mouse offspring. Am J Transl Res. (2018) 10:975–88. 29636887 PMC5883138

[48] Sampino S Stankiewicz AM Zacchini F Goscik J Szostak A Swiergiel AH. Pregnancy at advanced maternal age affects behavior and hippocampal gene expression in mouse offspring. J Gerontol Ser A Biol Sci Med Sci. (2017) 72:1465–73. 10.1093/gerona/glx016 28329103 PMC5861961

[49] Cimadomo D Fabozzi G Vaiarelli A Ubaldi N Ubaldi FM Rienzi L. Impact of maternal age on oocyte and embryo competence. Front Endocrinol. (2018) 9:327. 10.3389/fendo.2018.00327 30008696 PMC6033961

[50] Fergusson DM Woodward LJ. Maternal age and educational and psychosocial outcomes in early adulthood. J Child Psychol Psychiatry Allied Discipl. (1999) 40:479–89. 10190348

