# Supplementary material for: Maternal age at childbirth and the risk of attention-deficit/hyperactivity disorder and learning disability in offspring
Source: Front Public Health. 2023 Feb 2;11:923133. doi: 10.3389/fpubh.2023.923133 (PMC9931903; doi:10.3389/fpubh.2023.923133)
Supplement: Supplementary file 1 [file Table_1.DOCX]

**Table S1.** Sensitivity analysis of the association between maternal age at childbirth and parent-reported LD, ADHD, and both LD and ADHD.

|  | ≤17 | 18–24 | 25–29 | 30–34 | 35–39 | ≥40 |
| --- | --- | --- | --- | --- | --- | --- |
| ADHD | 1.11 (0.71, 1.72) | 1.31 (0.98, 1.76) | 1 | 0.93 (0.59, 1.47) | 0.60 (0.34, 1.00) | 1.44 (0.49, 4.22) |
| LD | 1.72 (1.06, 2.78) | 1.33 (0.99, 1.78) | 1 | 1.39 (0.95, 2.05) | 1.30 (0.96, 1.78) | 2.12 (0.74, 6.04) |
| ADHD+LD | 1.71 (1.20, 2.43) | 1.46 (1.17, 1.82)* | 1 | 1.18 (0.83, 1.66) | 1.10 (0.82, 1.47) | 1.80 (0.73, 4.48) |

Model adjusted for age, gender, race, body mass index, poverty income ratio, smoking during pregnancy, and NHANES cycle.

We excluded subjects whitout data of body mass index or poverty income ratio.

* the P value was less than the Bonferroni adjustment (0.010).
